# Linking nighttime outdoor lighting attributes to pedestrians' feeling of safety: An interactive survey approach

**DOI:** 10.1371/journal.pone.0242172

**Published:** 2020-11-10

**Authors:** Boris A. Portnov, Rami Saad, Tamar Trop, Doron Kliger, Alina Svechkina

**Affiliations:** 1 Department of Natural Resources and Environmental Management, School of Environmental Studies, University of Haifa, Haifa, Israel; 2 Department of Economics, University of Haifa, Haifa, Israel; Tsinghua University, CHINA

## Abstract

Public space lighting (PSL) contributes to pedestrians’ feeling of safety (FoS) in urban areas after natural dark. However, little is known how different PSL attributes, such as illuminance, light temperature, uniformity and glare, affect people's FoS in different contextual settings. The present study aims to bridge this knowledge gap by developing a model linking different PSL attributes with FoS, while controlling for individual, locational, environmental and temporal factors. To develop such model, the study employs a novel interactive user-oriented method, based on a specially-designed mobile phone application–CityLights^TM^. Using this app, a representative sample of observers reported their impressions of PSL attributes and FoS in three cities in Israel, following a set of predetermined routes and points. As the study shows, higher levels of illumination and uniformity positively affect FoS, while lights perceived as warm tend to generate higher FoS than lights perceived as cold. These findings may guide future illumination polices aimed at promoting energy efficiency while ensuring urban sustainability.

## 1. Introduction

Public space lighting (PSL) is an important component of the urban environment and has a major contribution to the pedestrians’ overall feeling of safety (FoS) and comfort after natural dark [1–3]. Effectively, PSL is characterized by several attributes—illuminance, light color temperature, uniformity and glare [4],—each of which may influence FoS, as well as energy consumption [5–8], and human health [9, 10].

PSL is currently designed according to international, national or local standards, such as EN 13201 [11]. However, since the association between the above-mentioned lighting attributes and pedestrians’ FoS has not been sufficiently studied [1, 12], it is not clear enough how well do the abovementioned standards, albeit technically efficient, contribute to FoS. Moreover, human perceptions of PSL attributes may be influenced by socio-demographic characteristics and may vary according to individual, locational, environmental and temporal circumstances. Therefore, an *empirical model that links lighting attributes to FoS and considers various contextual factors is currently lacking*.

The present study attempts to fill this knowledge gap by *developing a PSL-FoS model that links illumination*, *light color temperature*, *uniformity and glare values with FoS*, *while controlling for individual*, *locational*, *environmental and temporal factors*. Such a model may help to adapt PSL to various settings affecting FoS, and thus may contribute to increased urban residents' satisfaction and wellbeing, while reducing health and ecological risks, and increasing energy efficiency.

Several studies have looked into the factors affecting the *perceived quality of nighttime illumination* by pedestrians [7, 13–24]. However, most of these studies were performed in laboratory conditions, which cannot realistically reflect the complexity of outdoor environmental factors (e.g., time, weather, vegetation density and terrain), thus potentially causing biases in the information collected.

Studies focusing on *outdoor* lighting constitute a distinct strand of research [7, 12, 25, 26], but they are relatively scarce and entail various limitations. Most of these studies are based on "self-report" questionnaires, which are subjects to various biases and limitations, such as inefficient management of information on measurement time and location during the assessments [27]. Using "paper & pencil" technique to record PSL assessments outdoor after-dark may also pose difficulties, especially to the elderly and visually impaired [28]. Consequently, some respondents may prefer to record their assessments later on, in better visual conditions, which may introduce uncontrolled errors, resulting from memory limitations [12].

Another bias may occur due to insufficient guidance to find the exact location of the designated assessment points. A significant bias may also result from having company during the assessment, such as in the studies of Kelly et al. [29] and Wu [2], where observers were sent to perform FoS assessments in couples or accompanied by research assistants.

In a recent study, Fotios & Johansson [23] examined how pedestrians appraise others' intentions in different outdoor settings. The researchers conclude that as long as a person can identify other pedestrians’ faces, PSL is sufficient. However, facial recognition, although important, may *not* be the sole factor that influences FoS, because while walking along a street, people often look down to avoid obstacles or intentionally avoid eye contact. Furthermore, the study in question was performed in a simulated built environment, and involved a relatively small number of subjects, which limits its generality.

To sum up, we should remark that previous studies that explored potential associations between artificial lighting and FoS have had several limitations. First and foremost, most of such studies were carried out in laboratory conditions [7]. Second, most of them used "paper & pencil" self-administered questionnaires, which entail well-known drawbacks, such as inefficient management of important information in a timely manner [30]. Third, most previous studies were based on a relatively small number of participants [14], which substantially limits their generality. Finally, none of the studies carried out to date attempted to *link different PSL attributes (especially light color temperature) with FoS*, *while controlling for individual*, *locational*, *environmental and temporal factors*.

The study, which results are described in the rest of this paper, attempts to address these limitations by exploring *how the abovementioned PSL attributes—illumination*, *light color temperature*, *uniformity and glare—affect pedestrians’ FoS in different contextual settings*.

## 2. Materials and methods

### 2.1 Data collection method

The study was approved by the ethics committee of the University of Haifa (Approval number 177/90) and participants' consent was obtained individually from each participant on-line, by the Dialog survey company, which hired survey participants and supervised data collection.

The study employs a novel interactive approach that uses a mobile phone application–CityLights^TM^,–which was designed by the authors and described in detail in our previous study, which solely focused on illumination [1]. The app was used by a representative sample of participants, who followed a set of predetermined routes and points, and reported their perceptions of PSL attributes and FoS, along with the date and time of assessments. The app was designed as location–and time-based, assuring that each participant reports *in situ* from the designated points, only after dark, and continuously follows a particular route without an option to report from the same point again. The following six measures were reported by each observer at each assessment point:

- Illumination (0 –very weak; 1 –reasonable; 2 –good; 3 –too strong);
- Light color temperature (0 –too cold;1 –a bit cold; 2 –a bit hot; 3 –too hot);
- Light uniformity (0 –non-uniform; 1 –slightly non-uniform; 2 –quite uniform; 3 –very uniform);
- Light glare (0 –not glaring; 1 –slightly glaring; 2 –quite glaring; 3 –very glaring);
- Feeling of safety (0 –feel very unsafe; 1 –feel little unsafe; 2 –feel reasonably safe; 3 –feel very safe).
- Overall lighting quality (0 –uncomfortable; 1 –slightly uncomfortable; 2 –quite comfortable; 3 –very comfortable).

This study focuses solely on FoS, mutually comparing it with PSL assessments. Investigation of other response variables, such as overall lighting quality, will be reported in future publications.

**Fig 1:**
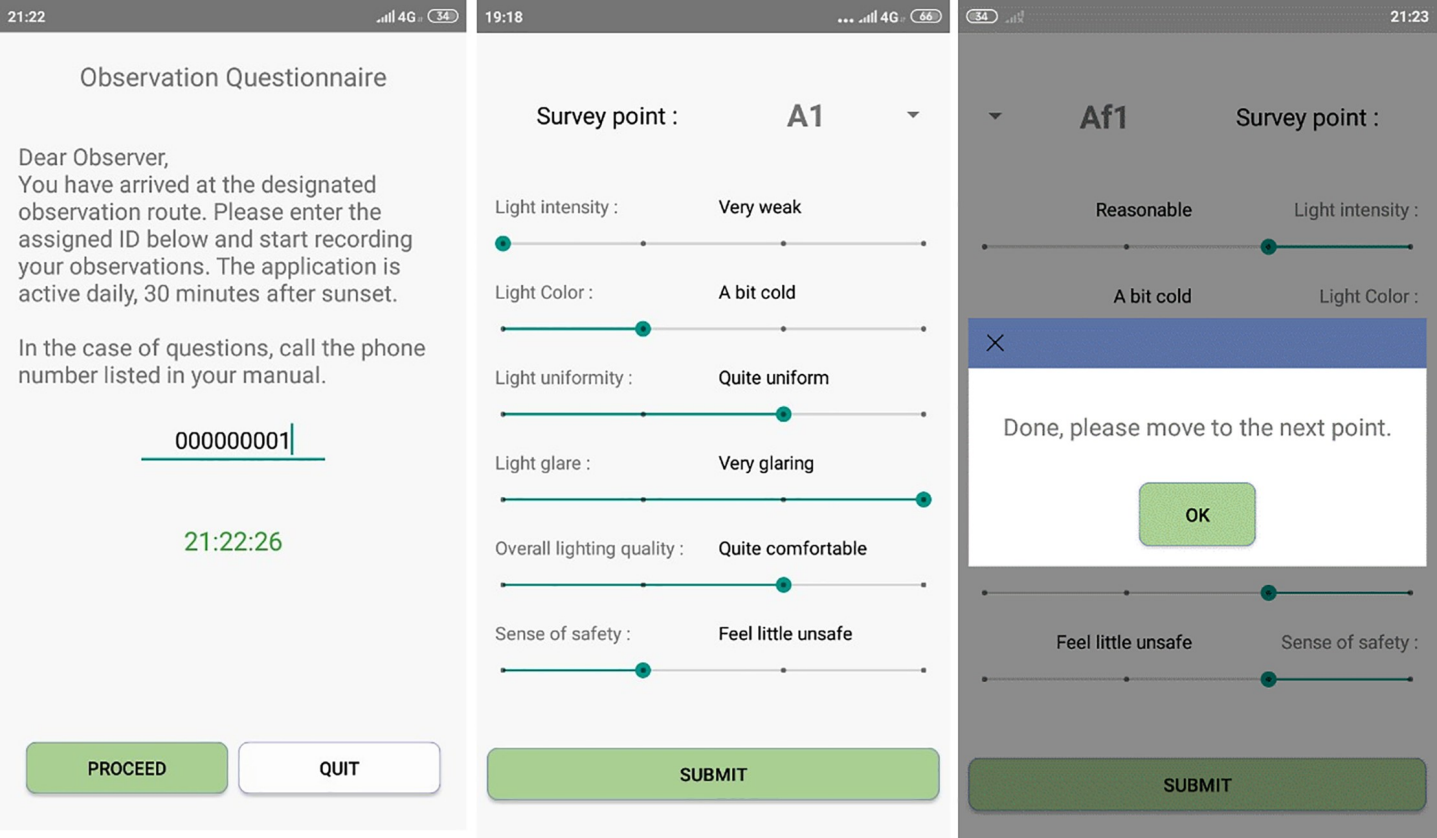
Screenshots of the CityLights^TM^ mobile application used to collect PSL and FoS assessments. A–entry screen; B–survey questions screen; C–direction screen.

The recorded assessments were uploaded in real time by each observer to the app cloud server, using a unique identification number designated beforehand to enter the app’s welcome screen. Fig 1 shows several screenshots of the app's interface.

### 2.2 Survey locations

The study was carried out in three large cities in Israel–Tel Aviv-Yafo, Haifa and Beersheba–along pre-defined routes in three-to-four neighborhoods in each city–altogether 10 routes, one in each neighborhood (see S1–S2 Appendices). Although the survey cities differ from each other in development patterns, climate, and population makeup (see S3 Appendix), all the selected neighborhoods are typical high-density residential areas with a spatial extent of about 1 km^2^.

In each neighborhood, a pedestrian route, stretching for about 800-1000m, was designed to cover main and secondary streets, as well as open spaces. Along the sidewalks of each route, about 20–30 survey points were selected at intervals of 20–30 m from each other (see S1–S3 Appendices).

### 2.3 Data management

Data for the present analysis were downloaded from the mobile phone app server on January 26^th^, 2020, and included 26,043 individual records, reported by 380 observers from 257 assessment points in all three cities under study. A total of 103 individual records (0.39%) were removed after screening for missing accuracy information of the observers’ locations (see Subsection 3.4), which resulted in 25,940 records for subsequent analysis. In addition to the reported assessments, the following data were collected: (a) the routes’ attributes (vegetation density and traffic volume), which were assessed by the researchers *in situ*; (b) socio-demographics of the observers (gender, age and education), which were obtained from the pre-survey questionnaires filled by the survey participants; and (c) geographic location of the observation appoints, as well as time and date, which were recorded automatically by the app.

Table 1 features the main research variables, while some descriptive statistics of the database are reported in S4-S7 Appendices. In particular, S4 Appendix reports frequency statistics of selected research variables; S5 Appendix shows demographic attributes of the observers (age group, gender), in compare to countrywide population; S6 Appendix presents the distribution of records by city and neighborhood; and S7 Appendix breakdowns the records into the reporting hours and months.

**Table 1:** Research variables, their reference categories and codding.

Variable	Group	Coding variable categories	Reference category
**Dependent variable**			
Feeling of safety (FoS)		0 = very unsafe; 1 = little unsafe; 2 = reasonably safe; 3 = very safe	Very safe
**PSL-related explanatory variables:**			
Illumination (IL)	***PSL***	0 = very weak (IL 0); 1 = reasonable (IL 1); 2 = good (IL 2); 3 = too strong (IL 3)	IL3
Light color temperature (LCT)	*-"-*	0 = too cold (LCT 0); 1 = a bit cold (LCT 1); 2 = a bit hot (LCT 2); 3 = too hot (LCT 3)	LCT3
Uniformity (LU)	*-"-*	0 = non-uniform (LU0); 1 = slightly non-uniform (LU1); 2 = quite uniform (LU2); 3 = very uniform (LU3)	LU3
Glare (LG)	*-"-*	0 = not glaring (LG0); 1 = slightly glaring (LG1); 2 = quite glaring (LG2); 3 = very glaring (LG3)	LG3
**Control variables**:			
Education (Edu)	***SOC***	Years of schooling	-
Age group (Age)	*-"-*	18–40 (Age1); 41–60 (Age2); 61+ (Age3)	Age1
Gender	*-"-*	Female; Male	Female
Country of birth	*-"-*	Israel; Europe; Other countries (Oth.Cou.)	Israel
Vegetation density	***ENV***	NV = no vegetation (trees and shrubs do not obscure street lights); MV = sparse vegetation (trees and shrubs partially obscure street lights); HV = dense vegetation (trees and shrubs significantly obscure street lights)	NV
Traffic intensity	*-"-*	ST = sparse traffic (less than 5 vehicles per 15 minutes); MT = medium traffic (5–10 vehicles per 15 minutes); HT = intensive traffic (more than 10 vehicles per 15 minutes)	ST
City	***CITY***	TA = Tel Aviv-Yafo; HA = Haifa; BS = Beersheba	TA
Month	***TEM***	Aug = August; Sep = September; Oct = October; Nov = November; Dec = December; Jan = January; Feb = February	Aug
Measurement time (MT)	*-"-*	MT1 = Before 20.00; MT2 = between 20.00–22.00; MT3 = after 22.00	MT1

### 2.4 Positioning accuracy

To ensure that FoS and PSL assessments are performed at the predefined locations, an accuracy measure was monitored by mobile phones' GPS. Deviation was measured by the Euclidian distance between the point at which an observer has actually performed the assessments and the exact location of the designated point (see S8 Appendix). As well established, mobile GPS accuracy might be affected by several technical and environmental factors, including proximity to the signal source, the type of the cellular device, and personal smartphone settings, as well as buildings height and vegetation density. However, post-survey evaluation showed that the average accuracy of the observers’ actual reporting locations was 12.26 ± 0.08 (mean ± SE) meters, which is within the mobile phone GPS accuracy range (cf. e.g., [31]). This indicates that the observers, who were guided to the assessment points by detailed written instructions, were quite accurately at the designated places during reporting. To reduce the possibility of misalliance between the designated and actual assessment points even further, individual reports with missing accuracy records were excluded from the analysis.

### 2.5 Statistical modelling

The variable of interest–FoS–is an ordinal variable, measured, as previously mentioned, on the following 4-point Likert scale: feel very unsafe (0), feel little unsafe (1), feel reasonably safe (2), feel very safe (3). Therefore, the *ordered logistic regression* [32] was employed for the analysis. In this regression, explanatory variables (or predictors) "push" the dependent variable, either upward or downward, to adjacent categories [33, 34].

To identify the impact of different PSL attributes on FoS, we used PSL attributes and controls (see S4 Appendix) as predictors for FoS in a set of nested models. The first model included PSL attributes only. In the *second model*, we appended dichotomous city variables (dummies) to account for unobservable inter-city differences, not captured by other variables. Lastly, in the *third (augmented) model* we appended social demographic, temporal and environmental factors (see S4-S7 Appendices).

To run the models, we coded the categorical variables as indicators, i.e., assigned the value of 1 if the category of interest is compatible with the observation (otherwise 0), thus making it possible to test the effect of a specific category, such as the city in which the measurement was taken, on the regression results [35]. The estimated models are represented by the following equations:

$$Model\;1:\;y_{i}=\alpha j+\beta *\;PSL_{lsi}$$ (1)

$$Model\;2:\;y_{i}=\alpha j+\beta *\;PSL_{lsi}+\gamma *\;CITY_{i}$$ (2)

$$Model\;3:\;y_{i}=\alpha j+\beta *\;PSL_{lsi}+\gamma *\;CITY_{i}+\delta *\;SOC_{is}+\eta *\;ENV_{il}+\theta *\;TEM_{i},$$ (3)

where:

y_i_ = $\log\frac{p(FoS\leq j)}{p(FoS>j)}$ with $\frac{p(FoS\leq j)}{p(FoS>j)}$ the odds of FoS being less than or equal to a particular value category;

*i* (1, …, 25,940) = observations in the whole dataset;

*j* (0, …, 3) = assessment categories provided for each PSL variable;

*l* (1, …, 257) = assessment points;

*s* (1, …, 380) = observers;

*p* refers to probability;

*p*(*FoS≤j*) is the cumulative probability of FoS being less than or equal to a specific category, where j = 0, …, 3 since *p(FoS = 4)* = 0;

***PSL*** = vector of subjective PSL assessments, which include illumination, light color temperature, light uniformity and glare (see Table 1), while β is their vector of corresponding regression coefficients;

***CITY*** = vector of cities dummies (see Table 1); γ is their vector of corresponding regression coefficients;

***SOC*** = vector of socio-demographic attributes, which include age group, gender, country of birth and education (see Table 1); δ is their vector of corresponding regression coefficients;

***ENV*** = vector of environmental dummies, which include traffic intensity and vegetation density (see Table 1); *η* is their vector of corresponding regression coefficients;

***TEM*** = vector of temporal dummies, which include the month and time of the measurement (see Table 1); θ is their vector of corresponding regression coefficients; and α is vector of intercepts estimated by the models.

The analysis was performed in the open source "R" software, using its *polr* function from the "MASS" library.

## 3. Results

### 3.1 General trends

**Fig 2:**
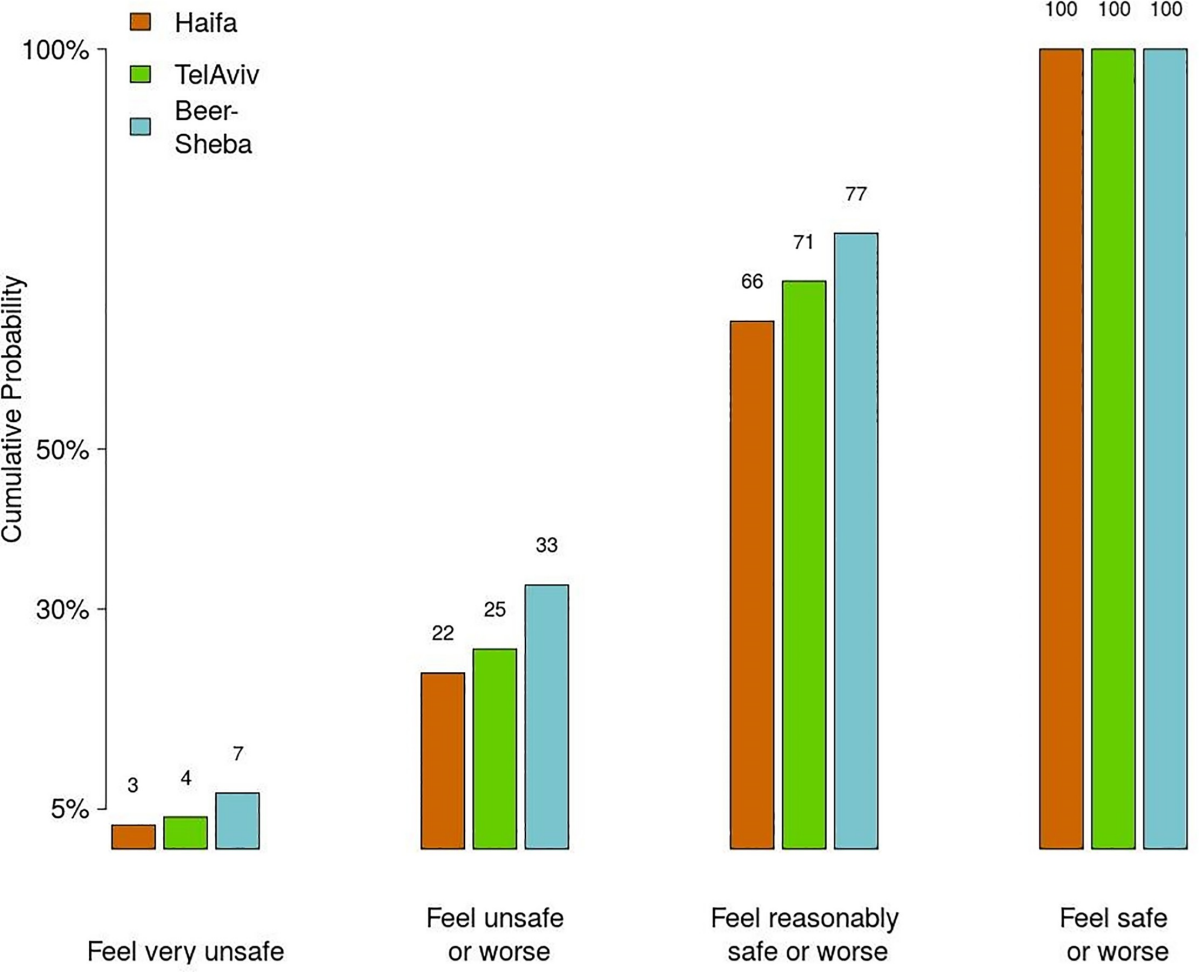
Cumulative probability of FoS assessments, as reported by the observers. Note: based on 25,940 individual reports.

**Fig 3:**
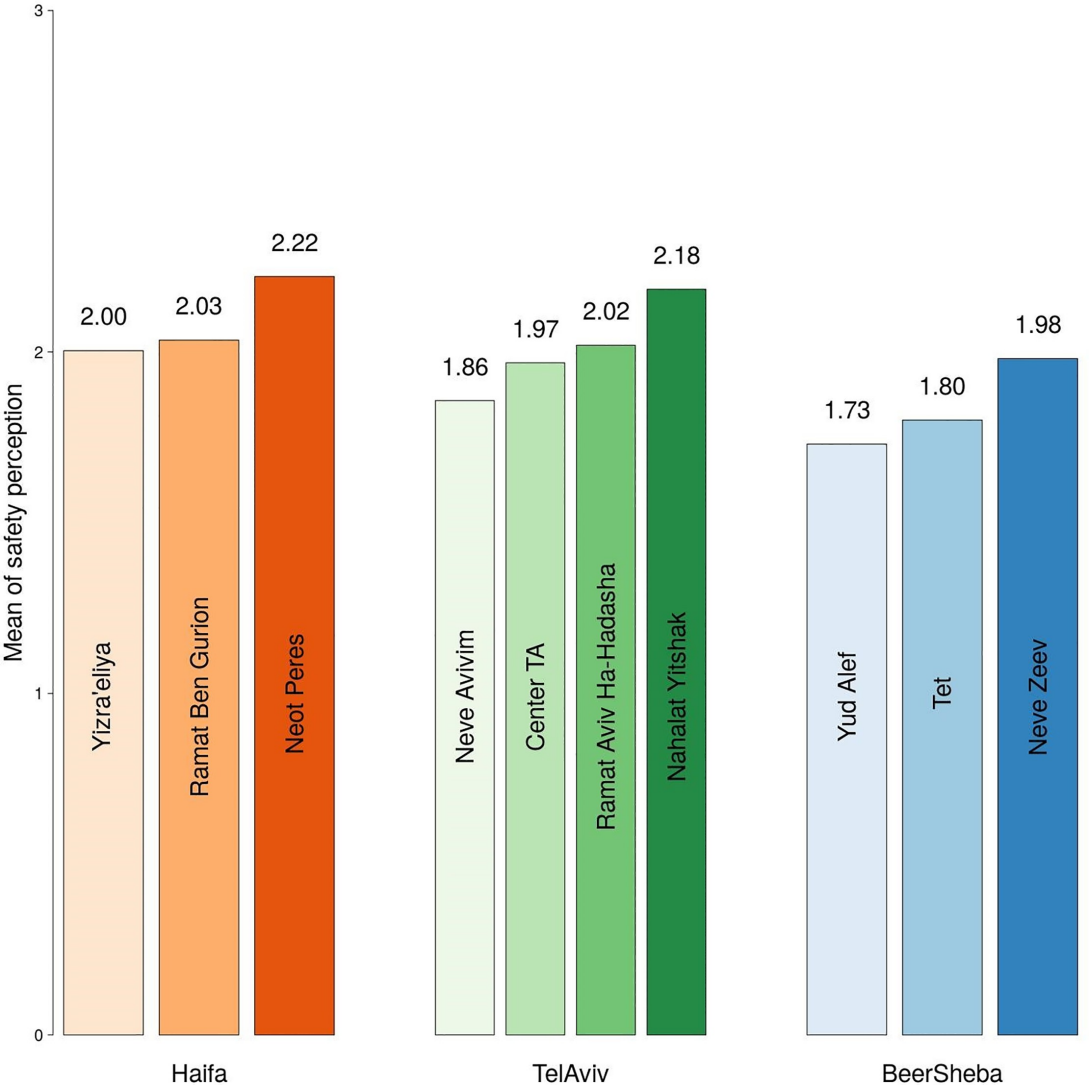
Mean values of FoS assessments in the neighborhoods surveyed. Note: Average values are reported on a four-point Likert scale from 0 (very unsafe) to 3 (very safe); for neighborhood maps and location, see S1-S2 Appendices.

Fig 2 shows the cumulative probabilities of *feeling unsafe* after dark in the three cities under study. The fig reveals that these probabilities are always the lowest in Haifa and the highest in Beersheba, and somewhere in between in Tel Aviv-Yafo. These findings indicate that under any given setting of PSL attribute, people *feel safer* in Haifa than in Tel Aviv-Yafo and Beersheba. Fig 3, which shows mean values of FoS assessments in all neighborhoods, reveals that the levels of FoS also differ substantially across neighborhoods, being highest in the "Neot Peres" neighborhood in Haifa (2.22 on a 4-point scale), and lowest in the "Yud Alef" neighborhood in Beersheba (1.73). Table 2, which reports results of the *X*^2^-test of statistical significance of differences in the observed FoS values, shows that the observed differences are highly significant (*X*^2^ = 310.08; p<0.01).

**Table 2:** *X*^2^-test of differences in the observed values of FoS across the cities surveyed (method: Pearson's *X*^*2*^ test).

City	Mean level of safety on a 4-point scale from 0 (very unsafe) to 3 (very safe)	*X*^*2*^ test (df = 6, 25940)
**Response variable: FoS**		
• Tel Aviv-Yafo	2.000	310.73***
• Haifa	2.080	
• Beer Sheba	1.837	

### 3.2 Regression models

**Fig 4:**
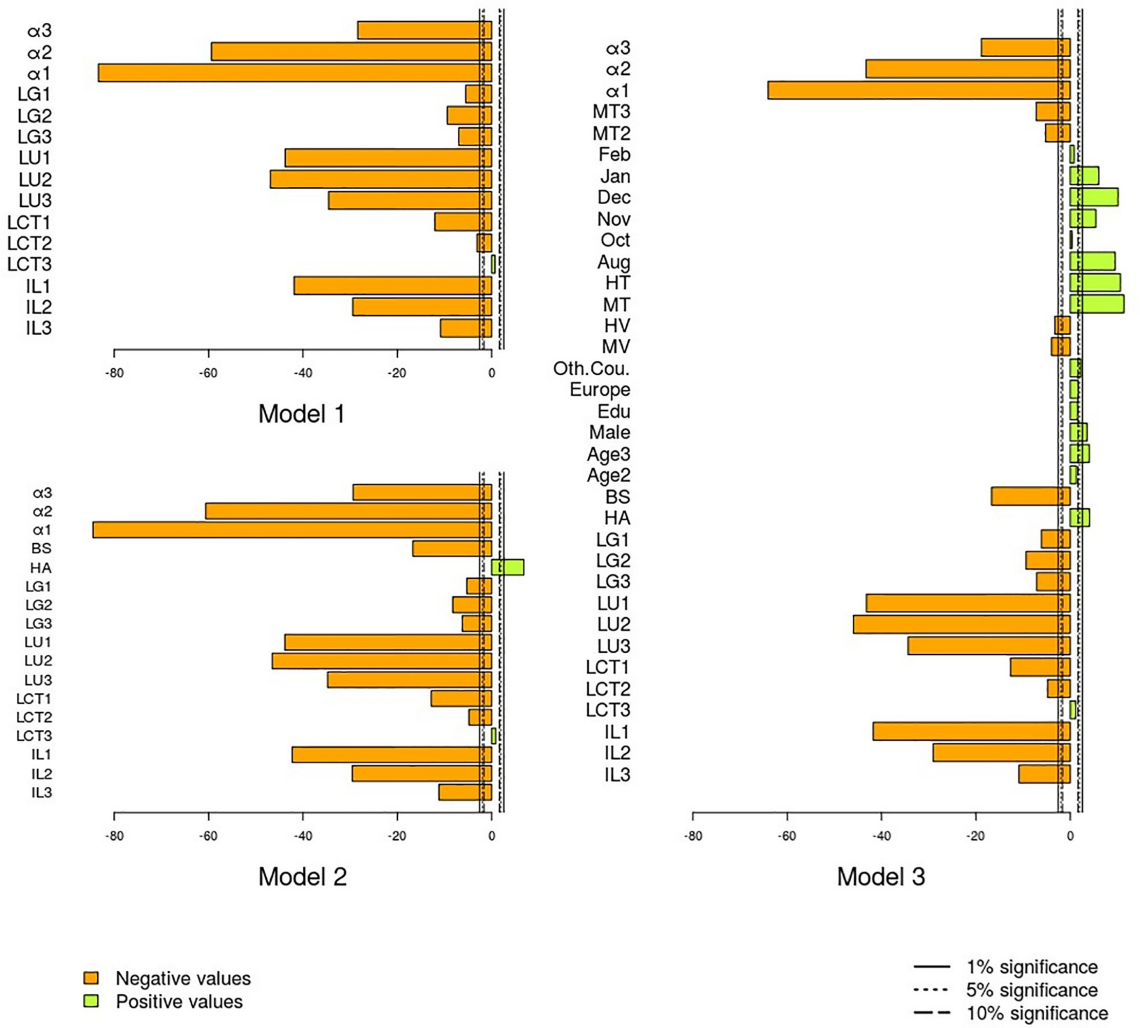
Significance levels of the research variables associated with FoS (*t*-stats, two-tailed; based on Models 1–3 in Tables 3–5). Note: see Table 1 for variables’ definitions.

Tables 3–5 present the estimated models. In particular, Table 3 shows results with PSL attributes only (Model 1); Table 4 –with PSL attributes and city dummies (Model 2); and Table 5 –with all research variables (Model 3). Fig 4 displays *t*-values for all three models together, to facilitate visual comparison. The orange bars in this Fig represent factors negatively effecting FoS, while the green bars represent factors positively affecting FoS.

**Table 3:** Factors affecting FoS in the neighborhoods surveyed (method: Multiple ordinary regression; dependent variable–FoS on a 4-point Likert scale from 0 (very unsafe) to 3 (very safe); Model 1: Only PSL attributes are included.

Predictor	B	*t*-stat	Exp(B)	95% CI	
				Lower	Upper
Illumination (ref. = too strong)					
• Good	-0.740	-10.824***	0.477	0.417	0.545
• Reasonable	-2.177	-29.423***	0.113	0.098	0.131
• Very weak	-3.802	-41.889***	0.022	0.019	0.027
Light color temperature (ref. = too hot)					
• A bit hot	0.048	0.715	1.049	0.920	1.195
• A bit cold	-0.211	-3.110***	0.810	0.709	0.925
• Too cold	-1.141	-12.040***	0.320	0.265	0.385
Uniformity (ref. = very uniform)					
• Quite uniform	-1.727	-34.557***	0.178	0.161	0.196
• Slightly non-uniform	-2.624	-46.898***	0.073	0.065	0.081
• Not uniform	-3.362	-43.784***	0.035	0.030	0.040
Glare (ref. = very glaring)					
• Quite glaring	-0.403	-6.982***	0.668	0.597	0.748
• Slightly glaring	-0.555	-9.397***	0.574	0.511	0.644
• Not glaring	-0.375	-5.507***	0.687	0.601	0.785
Intercepts					
• α1	-8.290	-83.349***			
• α2	-5.392	-59.382***			
• α3	-2.440	-28.380***			
Residual Deviance	46,511.89				
AIC	46,541.89				

***Notes*:** Ref. = reference category; Exp = exponent; CI = 95% confidence interval

*** indicates a 1% significance level (two-tailed)

** indicates a 5% significance level (two-tailed)

* indicates a 10% significance level (two-tailed).

**Table 4:** Factors affecting FoS in the neighborhoods surveyed (method: Multiple ordinary regression; dependent variable–FoS on a 4-point scale from 0 (very unsafe) to 3 (very safe); Model 2: City dummies added).

Predictor	B	*t*-stat	Exp(B)	95% CI	
				Lower	Upper
Illumination (ref. = too strong)					
• Good	-0.768	-11.191***	0.464	0.405	0.530
• Reasonable	-2.194	-29.567***	0.111	0.096	0.129
• Very weak	-3.849	-42.286***	0.021	0.018	0.025
Light color temperature (ref. = too hot)					
• A bit hot	0.055	0.831	1.057	0.927	1.204
• A bit cold	-0.329	-4.831***	0.719	0.629	0.822
• Too cold	-1.219	-12.799***	0.295	0.245	0.356
Uniformity (ref. = very uniform)					
• Quite uniform	-1.746	-34.771***	0.174	0.158	0.192
• Slightly non-uniform	-2.614	-46.515***	0.073	0.066	0.082
• Not uniform	-3.382	-43.851***	0.034	0.029	0.039
Glare (ref. = very glaring)					
• Quite glaring	-0.361	-6.224***	0.697	0.622	0.780
• Slightly glaring	-0.491	-8.263***	0.612	0.544	0.687
• Not glaring	-0.361	-5.276***	0.697	0.609	0.797
City dummies (ref. = Tel Aviv-Yafo)					
• Haifa	0.209	6.817***	1.232	1.161	1.309
• Beersheba	-0.528	-16.691***	0.590	0.555	0.628
Intercepts					
• α1	-8.448	-84.524***			
• α2	-5.506	-60.608***			
• α3	-2.518	-29.367***			
Residual Deviance	46,054.55				
AIC	46,088.55				

***Notes***: See notes to Table 3.

**Table 5:** Factors affecting FoS in the neighborhoods surveyed (method: Ordinary regression; dependent variable–perception of safety on a 4-point scale from 0 (very unsafe) to 3 (very safe); Model 3: City dummies, social demographic, temporal and locational variables added).

Predictor	B	*t*-stat	Exp(B)	95% CI	
				Lower	Upper
Illumination (ref. = too strong)					
• Good	-0.752	-10.858***	0.472	0.412	0.540
• Reasonable	-2.173	-29.056***	0.114	0.098	0.132
• Very weak	-3.820	-41.756***	0.022	0.018	0.026
Light color temperature (ref. = too hot)					
• A bit hot	0.077	1.148	1.080	0.947	1.233
• A bit cold	-0.331	-4.814***	0.718	0.627	0.821
• Too cold	-1.217	-12.677***	0.296	0.245	0.357
Uniformity (ref. = very uniform)					
• Quite uniform	-1.742	-34.354***	0.175	0.158	0.193
• Slightly non-uniform	-2.605	-45.966***	0.074	0.066	0.083
• Not uniform	-3.351	-43.186***	0.035	0.030	0.041
Glare (ref. = very glaring)					
• Quite glaring	-0.417	-7.122***	0.659	0.587	0.739
• Slightly glaring	-0.562	-9.354***	0.570	0.507	0.641
• Not glaring	-0.423	-6.113***	0.655	0.572	0.750
City dummies (ref. = Tel Aviv-Yafo)					
• Haifa	0.141	4.071***	1.152	1.076	1.233
• Beersheba	-0.582	-16.667***	0.559	0.523	0.598
Age interval dummies (ref. = 18–40)					
• 41–60	0.039	1.273	1.040	0.979	1.104
• 61+	0.203	4.019***	1.225	1.110	1.353
Gender dummy (ref. = female)					
• Male	0.093	3.565***	1.098	1.043	1.155
Education (number of years of schooling)	0.007	1.554	1.007	0.998	1.016
Country of birth dummies (ref. = Israel)					
• Europe	0.095	1.712*	1.099	0.986	1.224
• Other countries	0.118	2.218**	1.125	1.014	1.248
Traffic intensity (ref. = sparse traffic)					
• Average traffic intensity	0.342	11.379***	1.408	1.328	1.494
• Intense traffic	0.453	10.613***	1.573	1.447	1.710
Vegetation dummies (ref. = no vegetation)					
• Sparse vegetation	-0.116	-3.952***	0.891	0.841	0.943
• Dense vegetation	-0.142	-3.276***	0.868	0.798	0.945
Month dummies (ref. = September)					
• August	1.651	9.529***	5.214	3.723	7.344
• October	0.018	0.356	1.018	0.923	1.125
• November	0.238	5.427***	1.269	1.164	1.383
• December	0.408	10.114***	1.503	1.389	1.627
• January	0.315	6.037***	1.370	1.237	1.517
• February	0.114	0.775	1.121	0.842	1.496
Measurement time (ref. = before 20.00)					
• Between 20.00–22.00	-0.207	-5.226***	0.813	0.753	0.879
• After 22.00	-0.335	-7.236***	0.715	0.653	0.783
Intercepts					
• α1	-8.234	-64.045***			
• α2	-5.256	-43.279***			
• α3	-2.223	-18.838***			
Residual Deviance	45,527.95				
AIC	45,597.95				

***Notes*:** See notes to Table 3.

As Table 3 (Model 1) shows, *illumination* and *uniformity* significantly affect FoS, with *lower values* of these factors being *negatively* associated with *feeling safe* (Very weak light: Odds Ratio (OR) = 0.022; Non-uniform light: OR = 0.035; P<0.01). Results also indicate that lights with light color temperature *perceived as warm* are associated with *better* FoS (Too cold: OR = 0.320; p<0.01). Notably, *glaring light* is *positively* related to FoS (Not glaring (as compared to very glaring): OR = 0.687; p<0.01). Apparently, the observers perceive bright lights as beneficial, especially if they do not have to look at them directly.

Additional variables included in Model 2 and 3 (Tables 4–5), such as city dummies, vegetation dummies, traffic dummies, etc., emerge in the models as statistically significant (p<0.01). However, the inclusion of these additional variables into the models preserves the observed relationships between PSL attributes and FoS largely unchanged. This indicates that the observed relationships between PSL attributes and FoS are generally robust and *not altered* by socio-demographic, locational, environmental or temporal factors we considered.

In line with our initial results, the models show that the observers in Haifa have higher probability to feel safe than those in Tel Aviv-Yafo or Beersheba. In particular, compared to Tel Aviv-Yafo, odds ratios (ORs) for Haifa and Beersheba are 1.232 (p<0.01) and 0.590 (p<0.01), respectively (see Table 4).

Findings also show, *quite expectedly*, that male observers are more likely to feel safe in compare to females (OR = 1.098; p<0.01; see Table 5). However, rather unexpectedly, 61+yo appear to feel safer, compared to other age groups (OR = 1.225; p<0.01), probably due to reduced FoS among younger women. However, this linkage requires further analysis and can become a focus of future studies.

Regarding the impact of *country of birth* on FoS, the study reveals that, under a given level of illumination and all other factors held constant, observers born in Europe have a higher probability to feel safe than those born in Israel (OR = 1.099; 95%CI = 0.986, 1.224; p<0.1) (Table 5).

The impact of *vegetation density* and *traffic intensity* variables is also quite expected, with higher traffic intensity (that is, more dynamic lights associated herewith) increasing FoS and more obscuring vegetation decreasing it (P<0.01). Difference between assessments reported during *different hours of the evening* is also noteworthy. As the model indicates, observers who performed assessments after 22:00 are significantly *less* likely to feel safe, compared to observers who performed assessments before 20:00 (OR = 0.715; p<0.01), all other factors held constant.

Except for August, for which the small number of observations impairs generalization, there is a relatively clear trend of FoS increase during autumn and winter months in compare to the reference month–September (e.g., OR = 1.503 for December and OR = 1.370 for January (p<0.01); see Table 5). The cause for this trend is not sufficiently clear and needs to be investigated in future studies. However, it is reasonable to assume that this trend may be related to higher rates of street crime during warmer months [36].

**Fig 5:**
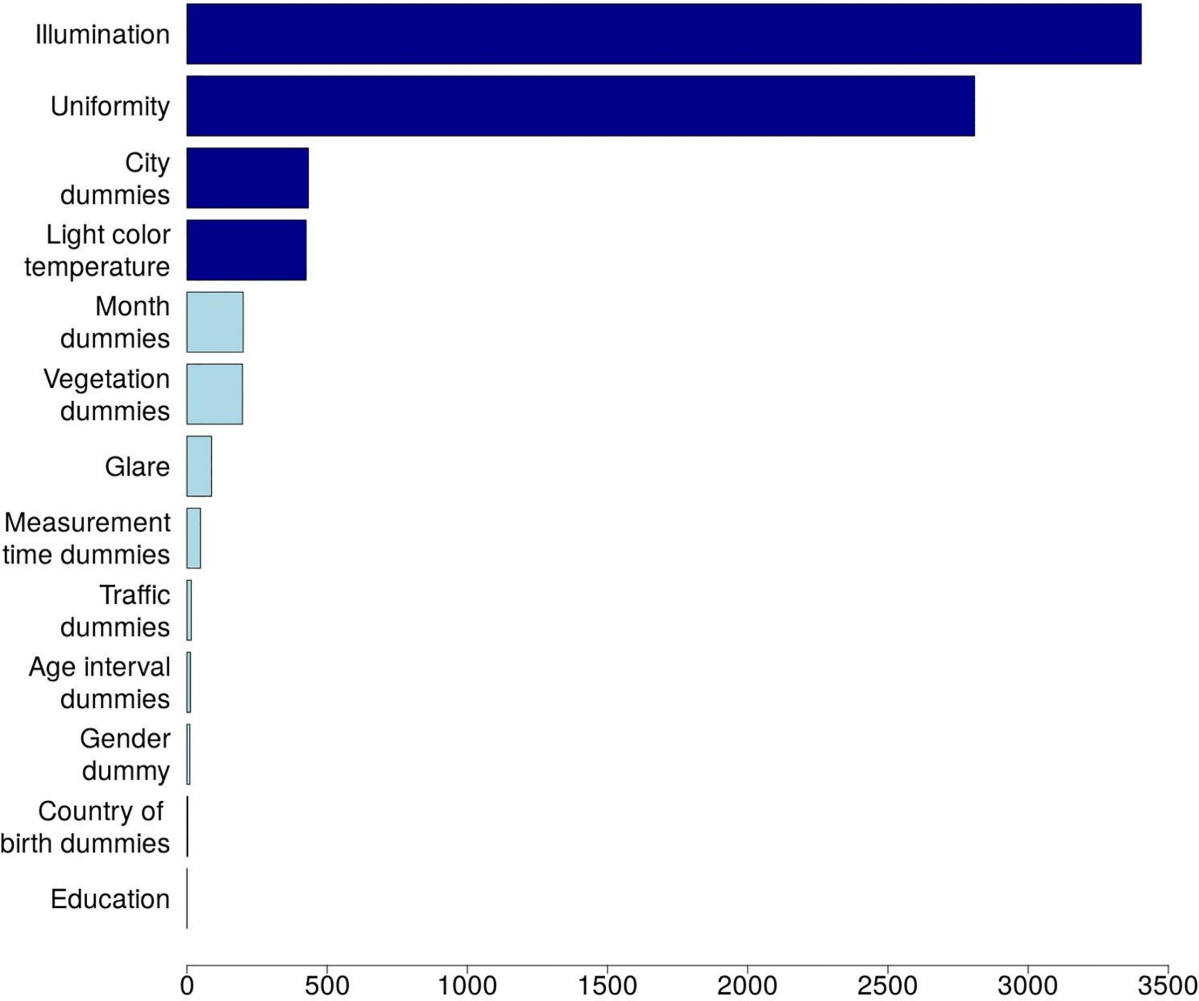
Relative contribution of different factors to FoS (the AIC increment test). Note: based on calculates reported in Table 6; see Table 1 for variables’ definitions; darker bars mark four most important variables, discussed in the text in some detail.

Table 6 and Fig 5 show results of the test, which ranks different *groups of factors* according to their contribution to the model. The factors ordering, which depicts diminishing contribution, indicates that FoS is mostly contributed to by *illumination* and *light uniformity*, followed by *city dummies* and *light color temperature*. This implies that none of these significant predictors can be removed from the augmented model without reducing its quality. As to education and the country of birth, their inclusion into the augmented model may be questioned.

**Table 6:** Test of the improvement in the model's fit attributed to the inclusion of different explanatory variables.

Category	AIC	AIC difference^a^	Probability, %^b^
Augmented model (all factors included)	45,597.95		
Illumination	49,001.25	3,403.30	0.0
Uniformity	48,407.12	2,809.17	0.0
City dummies	46,031.23	433.28	0.0
Light color temperature	46,023.14	425.19	0.0
Month dummies	45,798.56	200.61	0.0
Vegetation dummies	45,796.17	198.22	0.0
Glare	45,685.71	87.76	0.0
Measurement time dummies	45,646.50	48.55	0.0
Traffic intensity	45,613.51	15.56	0.0
Age interval dummies	45,610.24	12.29	0.2
Gender dummy	45,608.66	10.71	0.5
Country of birth dummies	45,601.15	3.20	20.2
Education	45,598.37	0.42	81.1

***Notes***: AIC = Akaike information criterion

^a^ AIC difference = difference between the AIC of the model missing one category to the AIC of the augmented model (the variables are listed in the order of diminishing contribution).

^b^ the probability ratio that a restricted model without a given category is as good as the augmented model, which includes all the explanatory variables.

### 3.3 Simulation test

**Fig 6:**
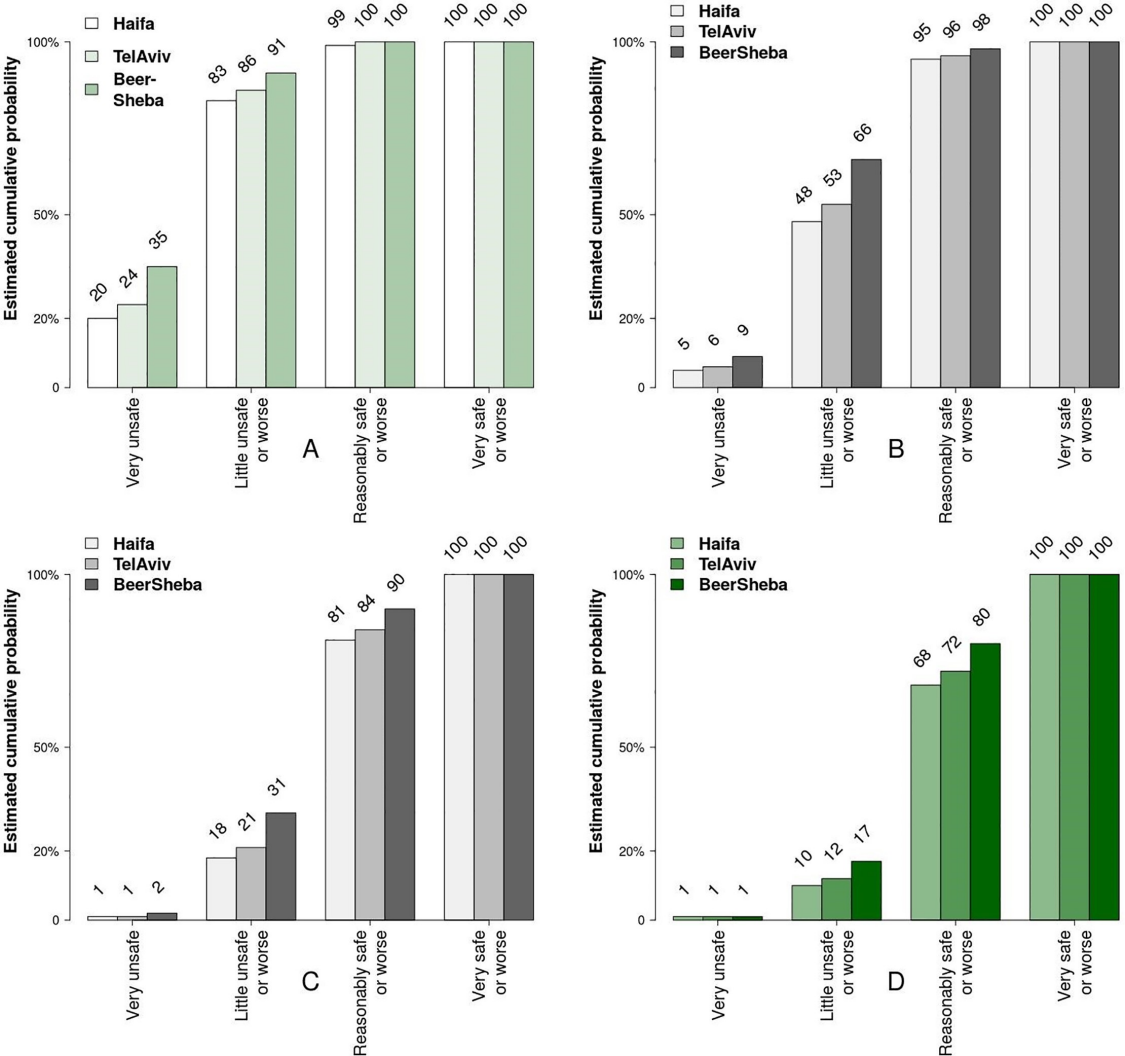
Cumulative probability estimates of FoS assessments for different cities and different levels of illumination (a model response test). Illumination levels: A = very weak; B = reasonable; C = good; D = strong. Note: The estimates are based on Model 2 (Table 4) and are generated by changing illumination levels only, while holding the values of all other variables constant and equal to 1 on a 4-point Likert scale (from 0 (Low) to 3 (High)).

**Fig 7:**
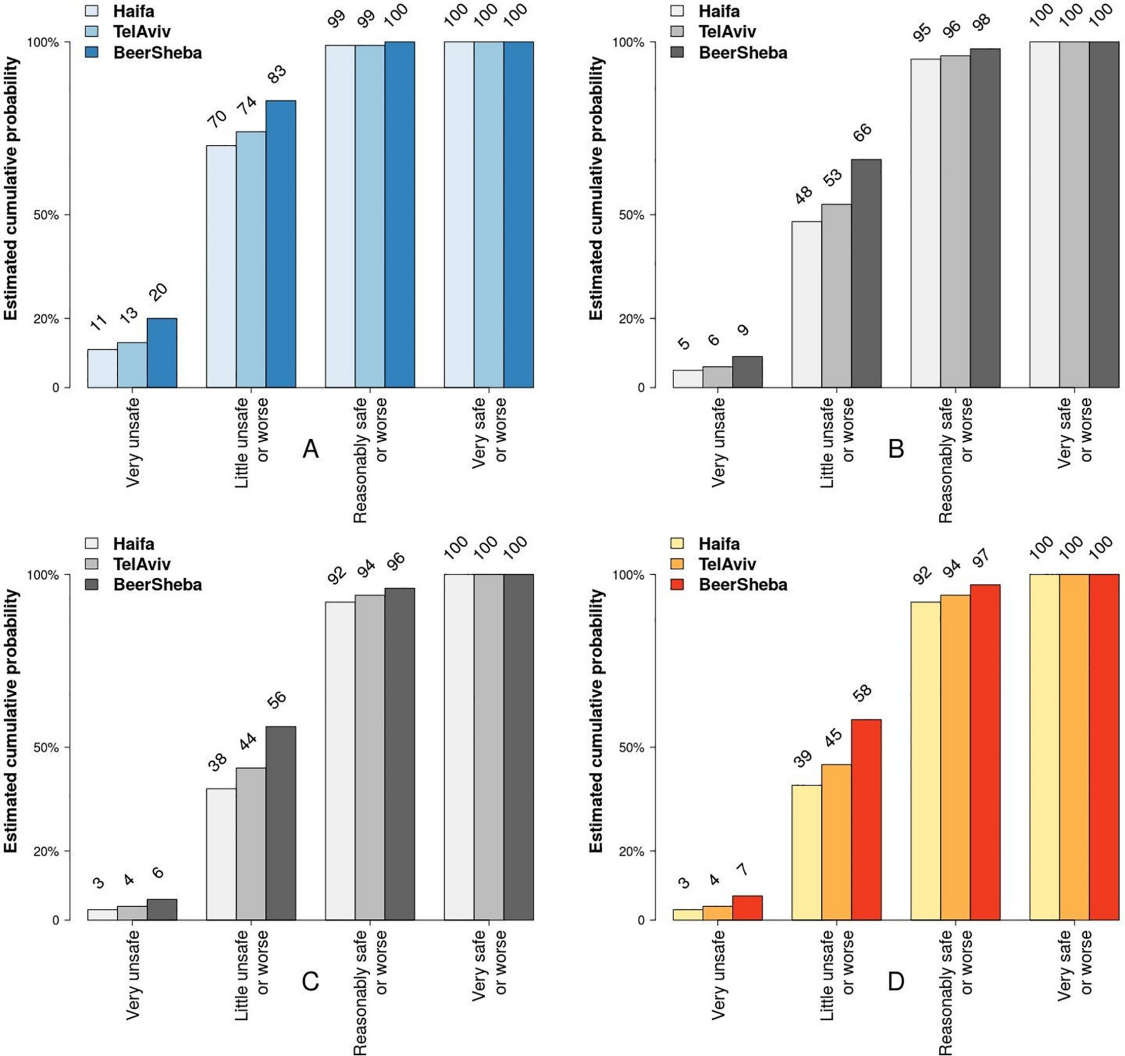
Cumulative probability estimates of FoS assessments for different cities and different light color temperatures (a model response test). Light temperature: A = too cold; B = a bit cold; C = a bit hot; D = too hot. Note: The estimates are based on Model 2 in Table 4 and are generated by changing light color temperature levels only, while holding the values of all other variables constant and equal to 1 on a 4-point Likert scale (from 0 (Low) to 3 (High)).

To estimate the response of FoS variable to different levels of *illumination* and *light color temperature*, our main variables of interest, we performed simulation tests on the estimated models by applying Model 2 (Table 4). In particular, using this model, we computed point estimates for various levels of these two variables. The tests were performed separately for each city under study, with of *all other* categorical variables set to *one* on their 4-point Likert scales. Figs 6 and 7 show test results as cumulative density functions (CDFs).

As Fig 6 shows, *if* illumination level is perceived as *low* (see Fig 6A), the probability of feeling *unsafe* is relatively high (i.e., 20% in Haifa, 24% in Tel Aviv-Yafo and 35% in Beersheba). Concurrently, if illumination level is perceived as *high* (see Fig 6D), the probability of feeling *unsafe* is less than 1% in all three cities, which indicates a fairly straightforward relationship between PSL and FoS. Moreover, under any given level of illumination, the cumulative probability of feeling *unsafe* is lowest in Haifa and highest in Beersheba, possible reasons for which we shall discuss later on.

As Fig 7 also shows, the probability of feeling *unsafe* (either very unsafe or little unsafe) is highest (70–83%), if lighting is perceived as cold (Fig 7A), and substantially lower (39–58%), all other factors held constant, if lighting is perceived as *warm* (Fig 6D). To put it differently, *warmer lights are associated with higher probability to feel safe*.

## 4. Discussion

The multivariate analysis of the recorded data revealed *several* important findings. First, it was found that locational, socio-demographic, temporal and environmental factors have a significant effect on FoS. Therefore, universal lighting standards *cannot be suitable to maintain the desired FoS in all urban areas*.

The analysis also shows that the minimal levels of illumination required for achieving the same level of FoS differ across cities, being higher in the inland arid city of Beersheba in compare to the coastal cities of Haifa and Tel Aviv-Yafo. This finding reconfirms results reported by Svechkina et al [1], which showed that residents of coastal areas, with often clouded skies and denser vegetation, may be satisfied with lower illumination intensity, in compare to residents of inland arid areas, characterized by stronger daytime lights and less light-obscuring vegetation.

Another interesting result stemming from the analysis is that *lights perceived as warm* tend to generate a better FoS than *colder lights*, given the same level of illumination. This finding may guide future illumination polices aimed at reducing unnecessary energy waste and preserving a cleaner urban environment.

Generally, our findings are in line with the results of Rahm [26], who demonstrates that PSL, along with individual and environmental factors, have a significant impact on pedestrians’ FoS. Nevertheless, the results of the current study are novel in several respects. First and foremost, improving pedestrians’ FoS can be reached either by switching the light color temperature from cold to warm, or, alternatively, by increasing illumination. The former approach is less energy consuming and may result in substantial economic and environmental benefits. Second, locational factors (such as different cities and neighborhoods), as well as temporal factors (such as hour of the evening and month of the measurements), which were not assessed in previous studies, were found in the present study to have a statistically significant effect on FoS.

It is important to note that in this study, the *predicted* FoS was linked to *assessed* PSL attributes, while, alternatively, the level of FoS could potentially be linked to *instrumental measurements*. Although both approaches–that is, individual assessments *vs*. instrumental measurements of PSL attributes–have their own merits, in the present study, we preferred to use the former assessment type. Our main reasoning is that people effectively link FoS to their assessment of lighting attributes, not to instrumental measurements, which they are unable to perform.

Lastly, we note that this study uses the standard ordered logistic model, which, at least in theory, may lead to erroneous conclusions that some explanatory variables are less significant than they actually are (false negative conclusions). However, since we have found significant effects, the issue is not major and the identified significance levels we have found may be viewed as a lower bound of the true significance. Future studies may consider the use of generalized ordered logistic models [37–39], to reduce this concern.

## 5. Conclusions

An explanatory model linking FoS with different PSL attributes, along with controlling for individual, locational, environmental and temporal factors, is estimated and reported in this study. To estimate this model, 380 observers were asked to assess PSL attributes in different urban settings during different hours of the evening, as well as to evaluate their perception of FoS associated herewith.

For this purpose, a specially designed cellular phone application, CityLights^TM^, which represents a novel interactive real-time methodological approach, was designed and employed. This app provides an efficient alternative to the "paper & pencil" questionnaires, commonly used to record observations made during an outdoor experience. Based on cloud technology and GPS, the app enables pre-authorized observers to record their assessments along with temporal and geographic information, while preventing observers from deviating too far from pre-determined reporting points. The app also prevents the same observer to enter the same assessment point more than once, and blocks the use of the app before the natural dark or by unauthorized people. In this manner, the app ensures the accuracy and integrity of assessments.

The present study is the second in a series of planned studies, carried as part of a large-scale research project, funded by the Israel Science Foundation. The project aims to model the perceived quality of PSL in urban areas by using an interactive user-oriented approach. The first paper in this sequence [1] aimed to identify only the *level of illumination* required by pedestrians to feel safe while walking through the streets at night. Concurrently, the present study links *different PSL attributes (illumination*, *light color temperature*, *uniformity and glare) to FoS*, *while controlling for individual*, *locational*, *environmental and temporal factors*.

The study was carried out in three large cities in Israel–Tel Aviv-Yafo, Haifa and Beersheba,–along pre-defined routes in 10 different neighborhoods (three-to-four neighborhoods in each city). A representative sample of participants used the CityLights^TM^ app to report their assessments from 257 pre-designated points, yielding altogether 25,940 individual valid records, which account for different PSL attributes.

The results of this study hold a potential for improving pedestrians’ FoS through better design of PSL by soliciting interactive responses from local residents and adjusting PSL attributes to their needs. Such a user-oriented approach allows to receive real-time feedback and may thus elevate PSL systems to a *new level of interactivity and intelligence*.

Findings of the study may also guide future PSL polices aiming at increasing energy efficiency and reducing health risks to humans and ecosystems. This goal can be achieved by adjusting not only illumination intensity, but also light color temperature, to the desired levels of FoS, concomitant with desired goals. By venue of this, the results presented in this paper can be of interest for urban planners, environmental law enforcement authorities and decision-makers in local municipalities. In particular, following the results presented in this paper, PSL design for desired FoS can enable temporal and locational variations of illumination intensity and light color temperature and their locational variation, to reflect population makeup and building patterns in different neighborhoods and in different cities, thus helping to achieve a desired level of FoS. In particular, study results may encourage PSL designers to use warmer light, which can generate higher FoS for the same level of illumination. Applying just this strategy may decrease PSL-associated costs, in compare to those generated by the commonly used cold lights. Furthermore, as warmer lights are associated with higher FoS, using such lights may compensate for a reasonable reduction in required illuminance, without compromising FoS. The model estimated and reported in this paper can help to estimate this possible tradeoff between required illumination level and light color temperature for any desired level of FoS.

The study findings may guide decision-makers towards improving new urban design and street retrofit projects. For instance, according to our findings, to achieve acceptable FoS levels, street lighting (SL) designers should focus on three *main* factors—illuminance, uniformity and light color temperature. In doing so, SL designers should keep in mind that, according to the study findings, urban residents appear to prefer warmer lights, which make them feel safer, in compare to cooler (i.e., bluer) lights. It is also important to keep in mind, that illumination levels should reflect contextual settings, considering that residents of cities, in which daytime radiance levels are high, may require more nighttime illumination to feel sufficiently safe after natural dark.

As the results of the present study also indicates, in designing PSL installations, planners should account for the demographics of potential users, such as age and gender, which were found in this study, along with the environmental characteristics of different urban settings, which appear to significantly affect FoS perceptions. Temporal factors should also be taken into account. For instance, PSL level should be designed to enable increased intensity in illumination or changing light color temperature towards warm after 22:00, in order to maintain the same level of FoS. Thus, the study may contribute to optimizing PSL standards, which are presently mostly geared towards road traffic rather than to pedestrians' needs.

Several interesting topics, which were beyond the scope of the present article, can be addressed in future studies. Two of them hold promising potential to quantify energy savings: the relation between FoS assessments and PSL instrumental measurements, and the potential linkage between illumination intensity and color temperature. Future studies may also increase the FoS-PSL model’s validity and credibility by using means, such as a larger number of observers in each neighborhood, additional neighborhoods in each city, additional cities, more diverse urban settings, and various socio-demographic and cultural conditions. Another important future direction for scholarly enquiry is *interactions* between different FoS predictors, such as e.g., age and gender, and their combined effects of FoS.

## Supporting information

S1 Appendix Location of the survey neighborhoods and survey routes in the cities under study. (DOCX)

S2 Appendix Nighttime lighting environment in selected neighborhoods under study. (DOCX)

S3 Appendix Physical, environmental and socio-demographic characteristics of the cities under study (as of 2018, unless stated otherwise)^a^. (DOCX)

S4 Appendix Frequency statistics of selected research variables (total number of individual reports = 25,940). (DOCX)

S5 Appendix Descriptive statistics of the survey participants compared to countrywide data. (DOCX)

S6 Appendix Number of observations, by city and neighborhood. (DOCX)

S7 Appendix Number of observations recorded during the survey and grouped by the time and month of observation (total = 25,940 individual reports). (DOCX)

S8 Appendix Accuracy of locational measurements, as assessed by the CityLights^TM^ survey application (total = 25,940 individual reports). (DOCX)
