# Supplementary material for: Linking nighttime outdoor lighting attributes to pedestrians' feeling of safety: An interactive survey approach
Source: PLoS One. 2020 Nov 10;15(11):e0242172. doi: 10.1371/journal.pone.0242172 (PMC7654807; doi:10.1371/journal.pone.0242172)
Supplement: S1 Appendix — (DOCX) [file pone.0242172.s001.docx]

**S1 Appendix:** Location of the survey neighborhoods and survey routes in the cities under study

A – Tel Aviv-Yafo

| 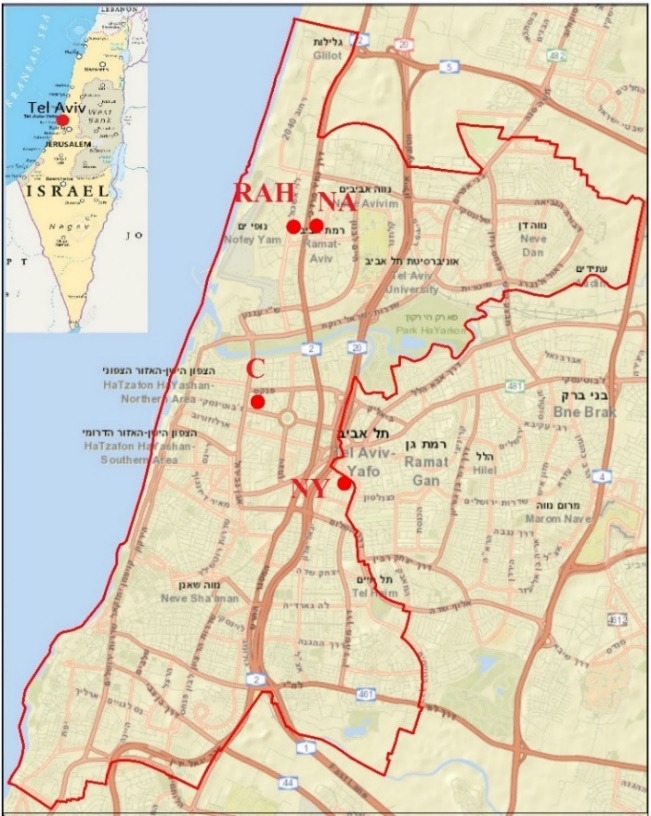 | 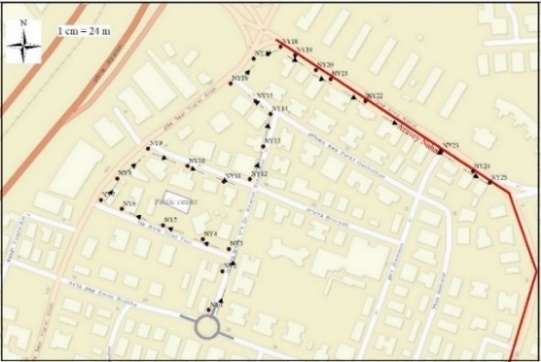  **NY** |
| --- | --- |
|  | (b) |
|  | 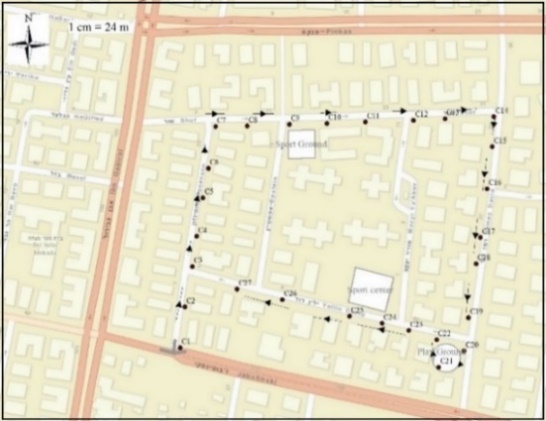  **C** |
| (a) | (c) |
| 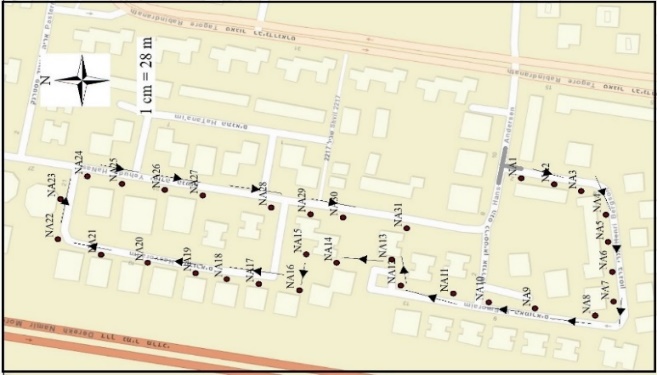  **NA** | 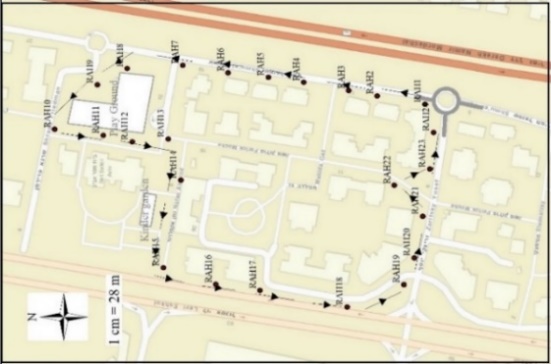  **RAH** |
| (d) | (e) |

(a) Neighborhoods surveyed: (b) “Nahalat Yitshak” (NY); (c) “HaTsafon HaHadash” (C); (d) “Neve Avivim” (NA); (e) “Ramat Aviv Ha-Hadasha” (RAH)

B – Haifa

| 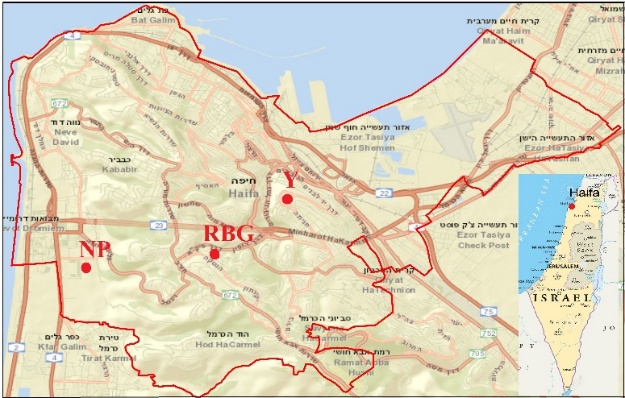 | 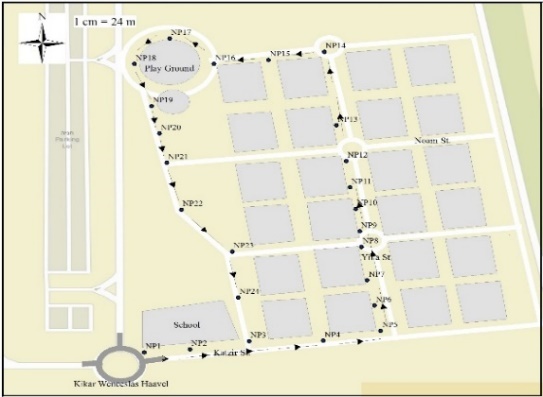  **NP** |
| --- | --- |
| (a) | (b) |
| 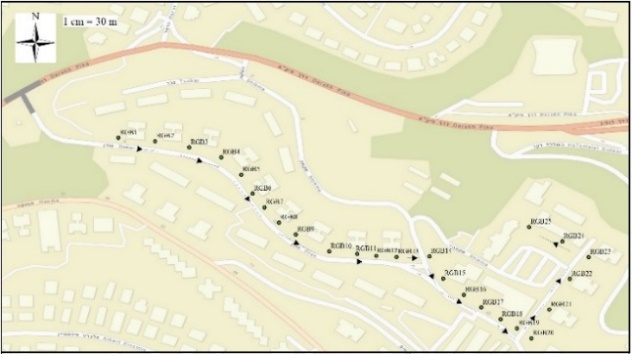  **RBG** | 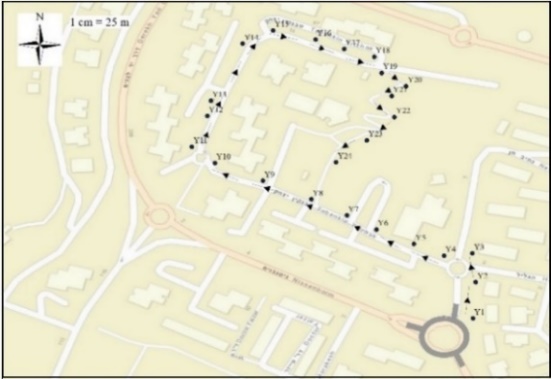  **Y** |
| (c) | (d) |

(a) Neighborhoods surveyed; (b) “Neot Peres” (NP); (c) ”Ramat Ben Gurion” (RBG); (d) “Yizra’eliya” (Y)

C- Beersheba

| 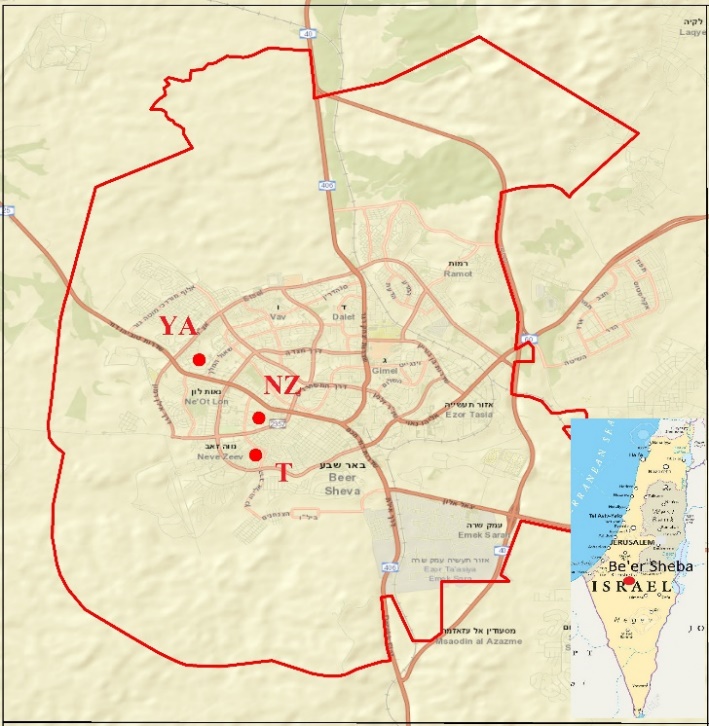 | 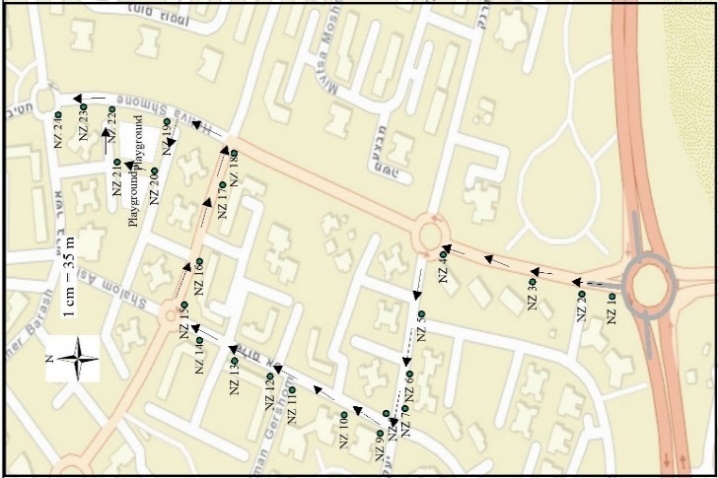  **NZ** |
| --- | --- |
| (a) | (b) |
| 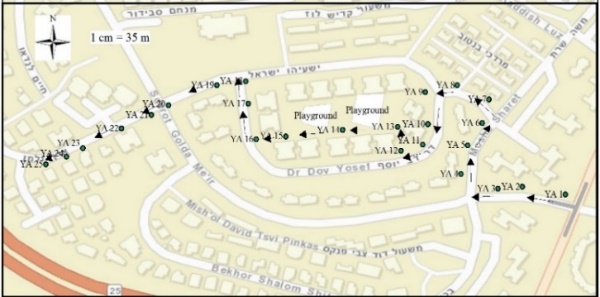  **YA** | 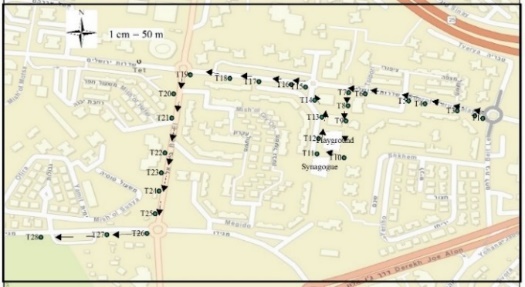  **T** |
| (c) | (d) |

(a) Neighborhoods surveyed; (b) “Never Ze’ev” (NZ); (c) “Yud-Alef” (YA); (d) “Tet” (T)
