# Supplementary material for: Linking nighttime outdoor lighting attributes to pedestrians' feeling of safety: An interactive survey approach
Source: PLoS One. 2020 Nov 10;15(11):e0242172. doi: 10.1371/journal.pone.0242172 (PMC7654807; doi:10.1371/journal.pone.0242172)
Supplement: S2 Appendix — (DOCX) [file pone.0242172.s002.docx]

**S2 Appendix:** Nighttime lighting environment in selected neighborhoods under study

| 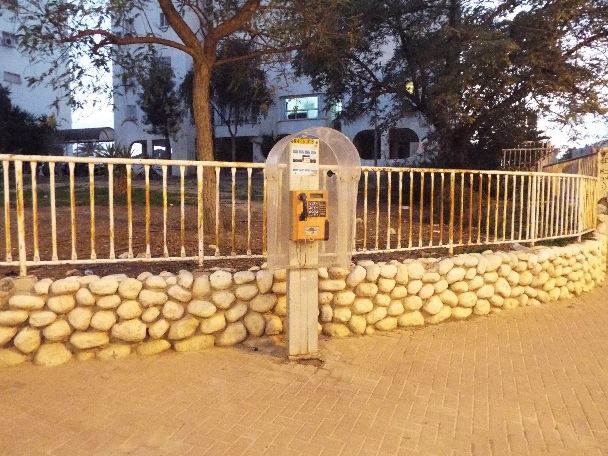 | 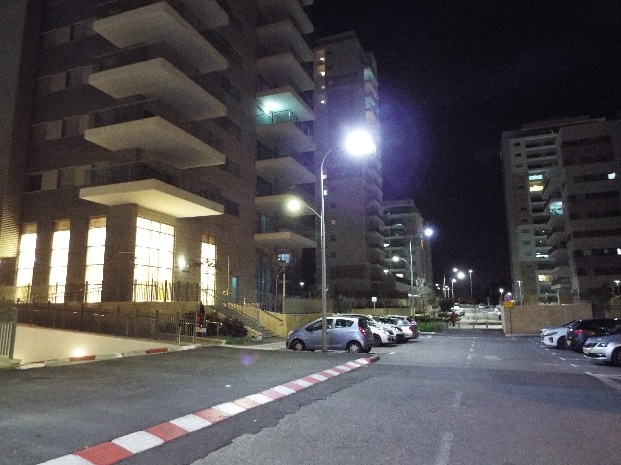 |
| --- | --- |
| A | B |
| 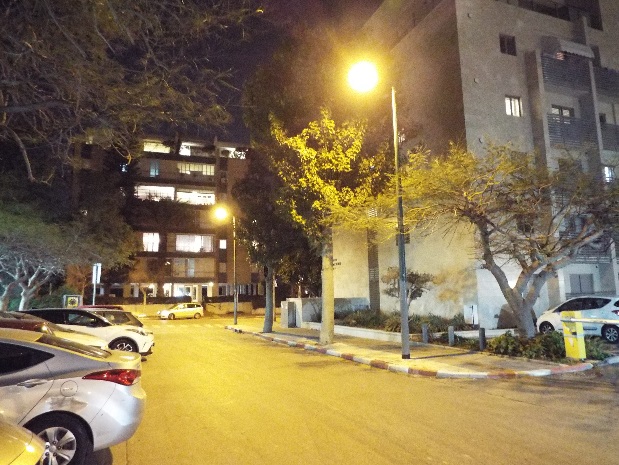 | 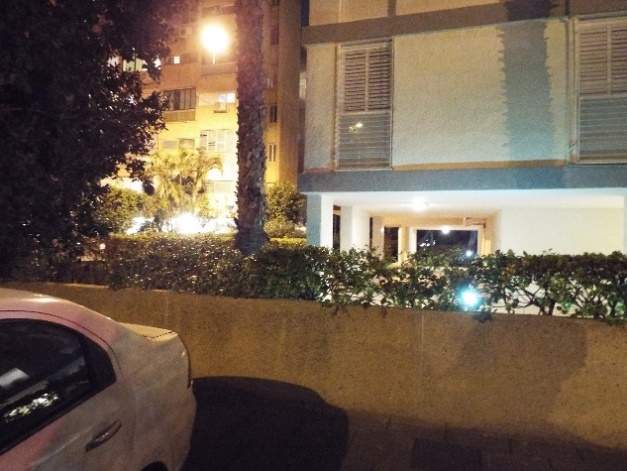 |
| C | D |

A – Neighborhood "Tet", Beer Sheba; B – neighborhood "Neot Peres", Haifa; C – neighborhood "HaTsafon HaHadash Center", Tel Aviv-Yafo; D – neighborhood "Ramat Aviv Ha-Hadasha", Tel Aviv-Yafo
