# Supplementary material for: Linking nighttime outdoor lighting attributes to pedestrians' feeling of safety: An interactive survey approach
Source: PLoS One. 2020 Nov 10;15(11):e0242172. doi: 10.1371/journal.pone.0242172 (PMC7654807; doi:10.1371/journal.pone.0242172)
Supplement: S3 Appendix — (DOCX) [file pone.0242172.s003.docx]

**S3 Appendix:** Physical, environmental and socio-demographic characteristics of the cities under study (as of 2018, unless stated otherwise)^a^

| **Development attribute** | **Locality** | | |
| --- | --- | --- | --- |
|  | **Tel Aviv-Yafo** | **Haifa** | **Be’er Sheba** |
| Population size (1,000) | 447.70 | 282.40 | 208.30 |
| Population density (per km^2^) | 8,354.70 | 4,045.50 | 1,733.20 |
| Climatic zone | [Mediterranean climate](https://en.wikipedia.org/wiki/Mediterranean_climate) | [Hot-summer Mediterranean climate](https://en.wikipedia.org/wiki/Hot-summer_Mediterranean_climate) | [Hot semi-arid climate](https://en.wikipedia.org/wiki/Hot_semi-arid_climate) with [Mediterranean](https://en.wikipedia.org/wiki/Mediterranean_climate)  influences |
| Average precipitation (mm) | 583.0 | 683.0 | 195.1 |
| Average precipitation days | 71.00 | 65.00 | 39.20 |
| Average relative humidity (%) | 67.00 | 69.00 | 42.00 |
| Country of birth (% for Jews) |  |  |  |
| - Israel | 69.00 | 52.00 | 55.0 |
| - Asia | 3.00 | 1.00 | 2.00 |
| - Africa | 16.00 | 22.00 | 22.0 |
| - Europe and America | 2.00 | 4.00 | 8.00 |
| Crime rates per 1,000 (2016), including^a^: | 3.60 | 3.05 | 4.76 |
| - Bodily harm | 0.62 | 0.63 | 0.86 |
| - Sexual offences | 0.05 | 0.05 | 0.07 |
| - Property offences | 0.67 | 0.70 | 0.74 |

^a^ Assembled or calculated using data from ICBS (2019).
