# Supplementary material for: Linking nighttime outdoor lighting attributes to pedestrians' feeling of safety: An interactive survey approach
Source: PLoS One. 2020 Nov 10;15(11):e0242172. doi: 10.1371/journal.pone.0242172 (PMC7654807; doi:10.1371/journal.pone.0242172)
Supplement: S4 Appendix — (DOCX) [file pone.0242172.s004.docx]

**S4 Appendix:** Frequency statistics of selected research variables (Total number of individual reports =25,940**)**

| Variable | Observation count | Percent |
| --- | --- | --- |
| **City** |  |  |
| - Tel Aviv-Yafo | 11,922 | 45.96 |
| - Haifa | 7,733 | 29.81 |
| - Beersheba | 6,285 | 24.23 |
| Total: | **25,940** | 100.00 |
| **Vegetation density** |  |  |
| - No vegetation | 120 | 46.69 |
| - Sparse vegetation | 107 | 41.63 |
| - Dense vegetation | 30 | 11.68 |
| Total: | **257** | 100.00 |
| **Traffic intensity** |  |  |
| - Sparse traffic | 148 | 57.59 |
| - Medium traffic | 82 | 31.91 |
| - Intensive traffic | 27 | 10.50 |
| Total: | **257** | 100.00 |
| **Age group** |  |  |
| - 18-40 | 223 | 58.68 |
| - 41-60 | 128 | 33.68 |
| - 61+ | 29 | 7.64 |
| Total: | **380** | 100.00 |
| **Gender** |  | 0.00 |
| - Female | 177 | 46.58 |
| - Male | 203 | 53.42 |
| Total: | **380** | 100.00 |
| **Country of birth** |  |  |
| - Israel | 330 | 86.84 |
| - Europe | 27 | 7.11 |
| - Other countries | 23 | 6.05 |
| Total: | **380** | 100.00 |
| **Month of observation** |  |  |
| - August 2019 | 173 | 0.67 |
| - September 2019 | 3,984 | 15.36 |
| - October 2019 | 3,082 | 11.88 |
| - November 2019 | 5,551 | 21.40 |
| - December 2019 | 9,593 | 36.98 |
| - January 2020 | 3,304 | 12.74 |
| - February 2020 | 253 | 0.98 |
| Total: | **25,940** | 100.00 |
| **Time of assessment (after sunset)** |  |  |
| - Before 20.00 | 4,059 | 15.65 |
| - Between 20.00 and 22.00 | 14,043 | 54.14 |
| - After 22.00 | 7,838 | 30.22 |
| Total: | **25,940** | 100.00 |
| **Perception of safety** |  |  |
| - Feel very safe (Level 4) | 412 | 5.65 |
| - Feel reasonably safe (Level 3) | 1,658 | 22.73 |
| - Feel little unsafe (Level 2) | 3,275 | 44.90 |
| - Feel very unsafe (Level 1) | 1,949 | 26.72 |
| Total: | **25,940** | 100.00 |
| **Assessment of Illumination** |  |  |
| - Too strong | 1,708 | 6.58 |
| - Good | 13,675 | 52.72 |
| - Reasonable | 8,187 | 31.56 |
| - Very weak | 2,370 | 9.14 |
| Total: | **25,940** | 100.00 |
| **Assessment of Light temperature** |  |  |
| - Too hot | 1,456 | 5.61 |
| - A bit hot | 13,840 | 53.35 |
| - A bit cold | 9,517 | 36.69 |
| - Too cold | 1,127 | 4.34 |
| Total: | **25,940** | 100.00 |
| **Assessment of Light Uniformity** |  |  |
| - Very uniform | 3,423 | 13.20 |
| - Quite uniform | 12,423 | 47.89 |
| - Slightly non-uniform | 8,172 | 31.50 |
| - Non-uniform | 1,922 | 7.41 |
| Total: | **25,940** | 100.00 |
| **Assessment of Glare** |  |  |
| - Very glaring | 2,308 | 8.90 |
| - Quite glaring | 9,342 | 36.01 |
| - Slightly glaring | 10,770 | 41.52 |
| - Not glaring | 3,520 | 13.57 |
| Total: | **25,940** | 100.00 |

Notes: In additional variable included in the analysis was education. However, this variable is continuous and not included into this table.
