# Supplementary material for: Linking nighttime outdoor lighting attributes to pedestrians' feeling of safety: An interactive survey approach
Source: PLoS One. 2020 Nov 10;15(11):e0242172. doi: 10.1371/journal.pone.0242172 (PMC7654807; doi:10.1371/journal.pone.0242172)
Supplement: S5 Appendix — (DOCX) [file pone.0242172.s005.docx]

**S5 Appendix:** Descriptive statistics of the survey participants compared to countrywide data

| Variable | Survey participants | | Countrywide (%)^a^ |
| --- | --- | --- | --- |
|  | Count | % |  |
| **Male** | **203** | 53 | 48 |
| - 18-40 | 117 |  |  |
| - 41-60 | 68 |  |  |
| - 61+ | 18 |  |  |
| **Female** | **177** | 47 | 52 |
| - 18-40 | 106 |  |  |
| - 41-60 | 60 |  |  |
| - 61+ | 11 |  |  |
| **Total:** | **380** | 100 | 100 |

^a^ Source: ICBS (Israel Central Bureau of Statistics)
