# Supplementary material for: Linking nighttime outdoor lighting attributes to pedestrians' feeling of safety: An interactive survey approach
Source: PLoS One. 2020 Nov 10;15(11):e0242172. doi: 10.1371/journal.pone.0242172 (PMC7654807; doi:10.1371/journal.pone.0242172)
Supplement: S6 Appendix — (DOCX) [file pone.0242172.s006.docx]

**S6 Appendix:** Number of observations, by city and neighborhood

| **City/neighborhood** | Number of observation point | Observation count | % of total |
| --- | --- | --- | --- |
| **Tel Aviv-Yafo** |  | **11,922** | **46** |
| - Neve Avivim | 31 | 3,494 | 13.5 |
| - Center | 27 | 2,886 | 11.1 |
| - Nahalat Yitshak | 25 | 2,916 | 11.3 |
| - Ramat Aviv Ha-Hadasha | 24 | 2,626 | 10.1 |
| **Haifa** |  | **7,733** | **30** |
| - Ramat Ben-Gurion | 25 | 2,722 | 10.6 |
| - Neot Peres | 24 | 2,330 | 9 |
| - Yizra'eliya | 24 | 2,681 | 10.4 |
| **Beersheba** |  | **6,285** | **24** |
| - Yud Alef | 25 | 1,927 | 7.3 |
| - Neve Zeev | 24 | 2,025 | 7.7 |
| - Tet | 28 | 2,333 | 9 |
| **Total:** | **257** | **25,940** | **100** |
