# Supplementary material for: Linking nighttime outdoor lighting attributes to pedestrians' feeling of safety: An interactive survey approach
Source: PLoS One. 2020 Nov 10;15(11):e0242172. doi: 10.1371/journal.pone.0242172 (PMC7654807; doi:10.1371/journal.pone.0242172)
Supplement: S7 Appendix — (DOCX) [file pone.0242172.s007.docx]

**S7 Appendix:** Number of observations recorded during the survey and grouped by the time and month of observation (Total=25,940 individual reports)

**
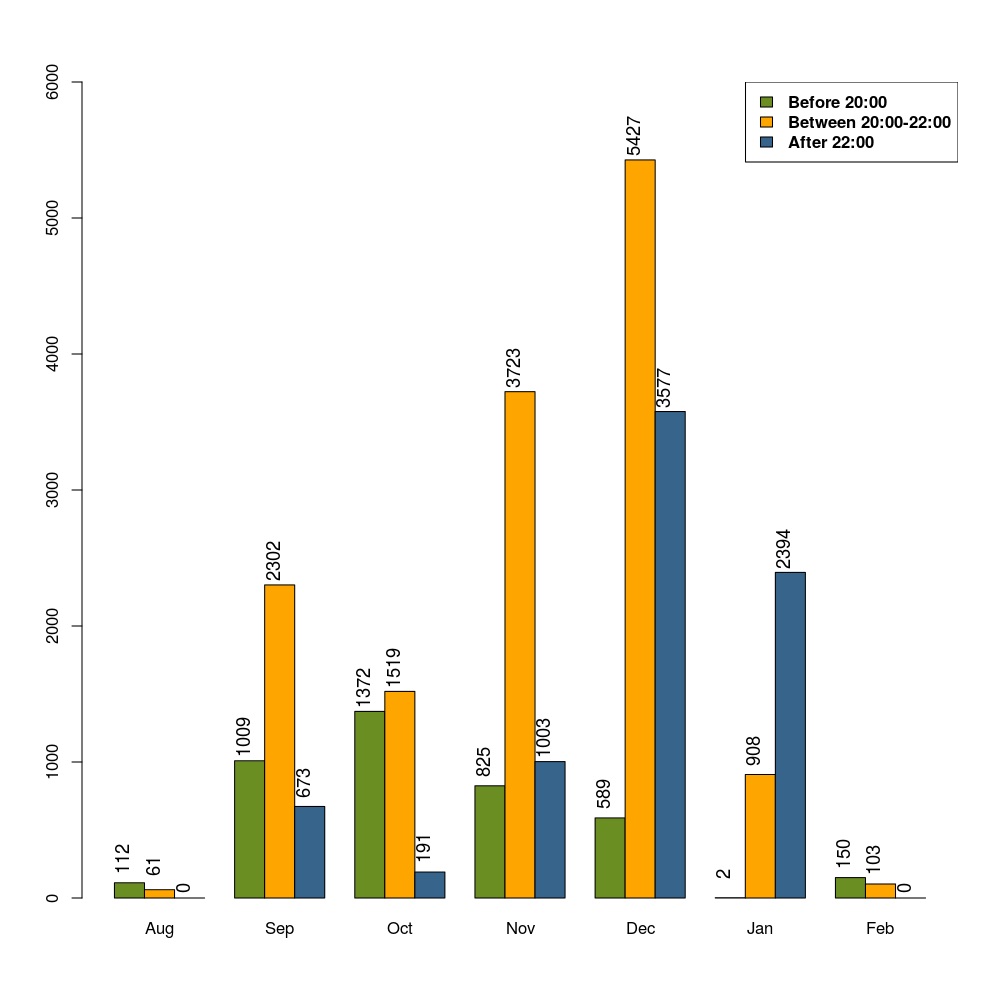
**
