# Supplementary material for: Linking nighttime outdoor lighting attributes to pedestrians' feeling of safety: An interactive survey approach
Source: PLoS One. 2020 Nov 10;15(11):e0242172. doi: 10.1371/journal.pone.0242172 (PMC7654807; doi:10.1371/journal.pone.0242172)
Supplement: S8 Appendix — (DOCX) [file pone.0242172.s008.docx]

**S8 Appendix:** Accuracy of locational measurements, as assessed by the CityLights^TM^ survey application (Total=25,940 individual reports)

**
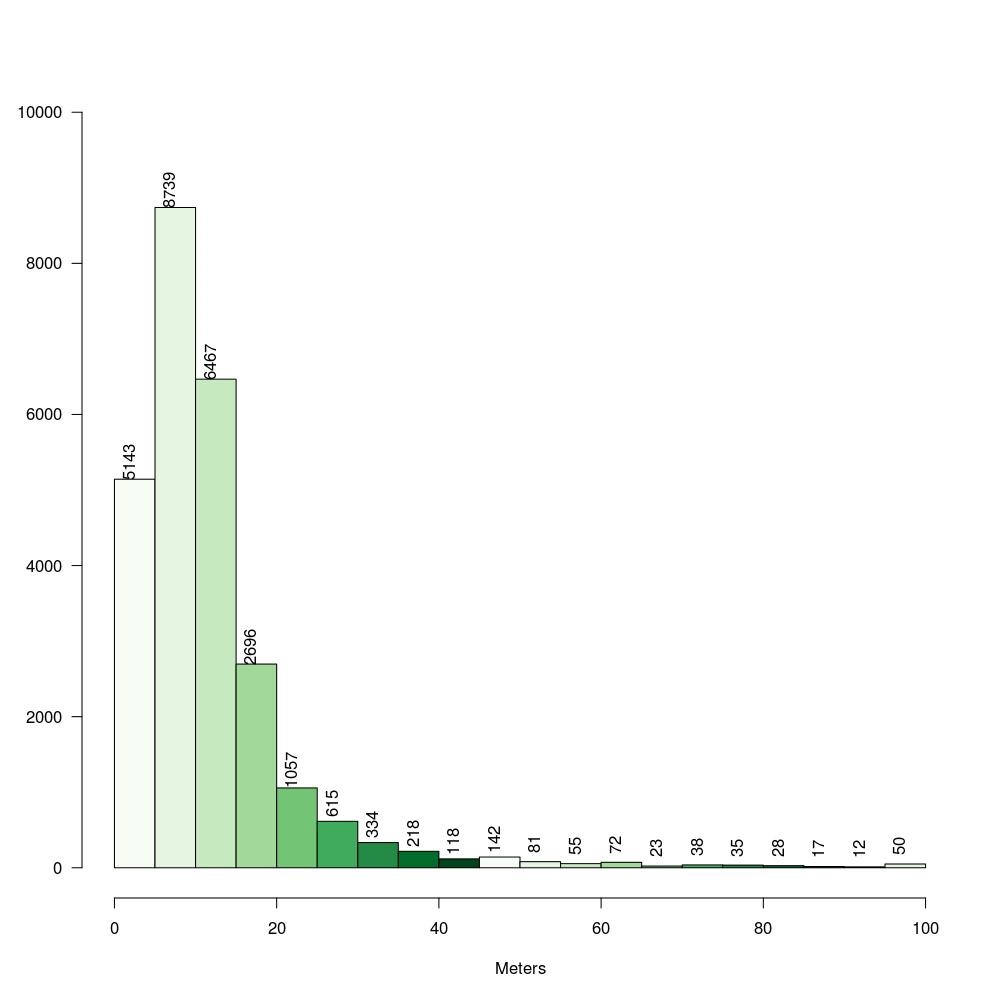
**
